# transcranial Direct Current Stimulation (tDCS) for the treatment and investigation of Phantom Limb Pain (PLP)

**DOI:** 10.1093/psyrad/kkac004

**Published:** 2022-04-22

**Authors:** Shahrzad Damercheli, Malin Ramne, Max Ortiz-Catalan

**Affiliations:** Center for Bionics and Pain Research, Mölndal 43130, Sweden; Department of Electrical Engineering, Chalmers University of Technology, Gothenburg 41296, Sweden; Center for Bionics and Pain Research, Mölndal 43130, Sweden; Department of Electrical Engineering, Chalmers University of Technology, Gothenburg 41296, Sweden; Center for Bionics and Pain Research, Mölndal 43130, Sweden; Department of Electrical Engineering, Chalmers University of Technology, Gothenburg 41296, Sweden; Operational Area 3, Sahlgrenska University Hospital, Mölndal 43180, Sweden; Department of Orthopaedics, Institute of Clinical Sciences, Sahlgrenska Academy, University of Gothenburg, Gothenburg 41345, Sweden

**Keywords:** Neuropathic pain, phantom limb pain, noninvasive brain modulation, transcranial direct current stimulation, pain rehabilitation, brain imaging, guided plasticity therapy

## Abstract

Phantom limb pain (PLP) is a complex medical condition that is often difficult to treat, and thus can become detrimental to patients’ quality of life. No standardized clinical treatments exist and there is no conclusive understanding of the underlying mechanisms causing it. Noninvasive brain stimulation (NIBS) has been used to find correlations between changes in brain activity and various brain conditions, including neurological disease, mental illnesses, and brain disorders. Studies have also shown that NIBS can be effective in alleviating pain. Here, we examined the literature on a particular type of NIBS, known as transcranial direct current stimulation (tDCS), and its application to the treatment of PLP. We first discuss the current hypotheses on the working mechanism of tDCS and then we examine published evidence of its efficacy to treat PLP. We conclude this article by discussing how tDCS alone, and in combination with brain imaging techniques such as electroencephalography (EEG) and magnetic resonance imagining, could be applied to further investigate the mechanisms underlying PLP.

## Introduction

Pain is one of the main components of protective reflexes of the human body (Cervero, 2012). Acute pain is usually related to damage to the body tissues, and thus has a protective and beneficial role by alerting the individual to a dangerous situation (e.g. touching a burning hot pot). However, experiencing pain is undesirable when there is no potential tissue damage, such as in the case of neuropathic pain that is pain arising due to a lesion or disease affecting the somatosensory system (IASP, 2021). Phantom limb pain (PLP), a type of neuropathic pain perceived as arising from a missing limb, is one of the most common problems faced by amputees (Katleho *et al*., 2020). PLP is challenging to treat and no standard clinical treatment exists. Available treatments of PLP can be classified into pharmaceutical, surgical, and clinical therapy methods (Malone and Strube, 1988). However, side effects of the first two methods have been a controversial issue, which has led scientists to investigate noninvasive clinical therapies with higher efficacy and fewer side effects. Clinical therapies for the treatment of PLP include physical and psychological therapies, plasticity-guided therapies, and noninvasive brain stimulation (NIBS), among others (Limakatso and Parker, 2021). NIBS includes techniques for stimulating or modulating brain activities without physical intrusion through the skin (Albizu *et al*., 2019). According to the stochastic entanglement hypothesis, brain stimulation can potentially facilitate the reconditioning of impaired sensorimotor neural networks when in combination with plasticity-guided therapies such as phantom motor execution and sensory training (Ortiz-Catalan, 2018), i.e. NIBS facilitates plasticity in plasticity-guided therapies. Furthermore, the use of NIBS alone had been shown to be beneficial for PLP relief, in particular for transcranial direct current stimulation (tDCS) (Bolognini *et al*., 2013; Bolognini *et al*., 2015). Here, we reviewed current hypotheses on the working mechanism of tDCS, the evidence for the efficacy of tDCS as a treatment of PLP, and the role that such method could have as a neuroscientific tool for investigating PLP.

## Mechanisms of Function Underlying the Effects of tDCS

Brain modulation can be top-down, where the stimulation is applied at the level of the central nervous system (CNS), or in the opposite direction (bottom-up), where the stimulation is applied to the peripheral nervous system propagating further to the CNS. Transcutaneous electrical nerve stimulation aims to stimulate the peripheral nervous system (bottom-up). The alleged purpose is to activate analgesic processes in the CNS by stimulating nonnociceptive neurons at the site of the nerve injury or amputation (DeSantana *et al*., 2009). The site of injury or the stump must be in a relatively healthy condition for transcutaneous electrical nerve stimulation to be applicable. For instance, stimulation of an open wound or irritated skin at the site of stimulation should be avoided.

Top-down NIBS can be delivered by at least four methods: transcranial magnetic stimulation (TMS), transcranial electrical stimulation (tES), transcranial focused ultrasound, and transcranial photobiomodulation (Albizu *et al*., 2019). Of the aforementioned techniques, TMS and tES have been most investigated as methods to alleviate pain (Lefaucheur, 2016; Lefaucheur *et al*., 2020).

TMS is based on the phenomenon of electromagnetic induction. An electric current is passed through a wire in a closed-circuit coil to produce a magnetic field that induces an electric current at the targeted site of the brain, resulting in induced action potentials (Bolognini *et al*., 2009). Conversely, tES applies a mild electrical field over the brain cortex to modulate brain activity in the targeted area. The effects of tES varies depending on the modulation of the current used to generate the electrical field, such as direct current (tDCS), alternating current, and random noises. Each mode of stimulation affects brain excitability differently, as does the placement of the electrodes (Inukai *et al*., 2016).

**Figure 1:**
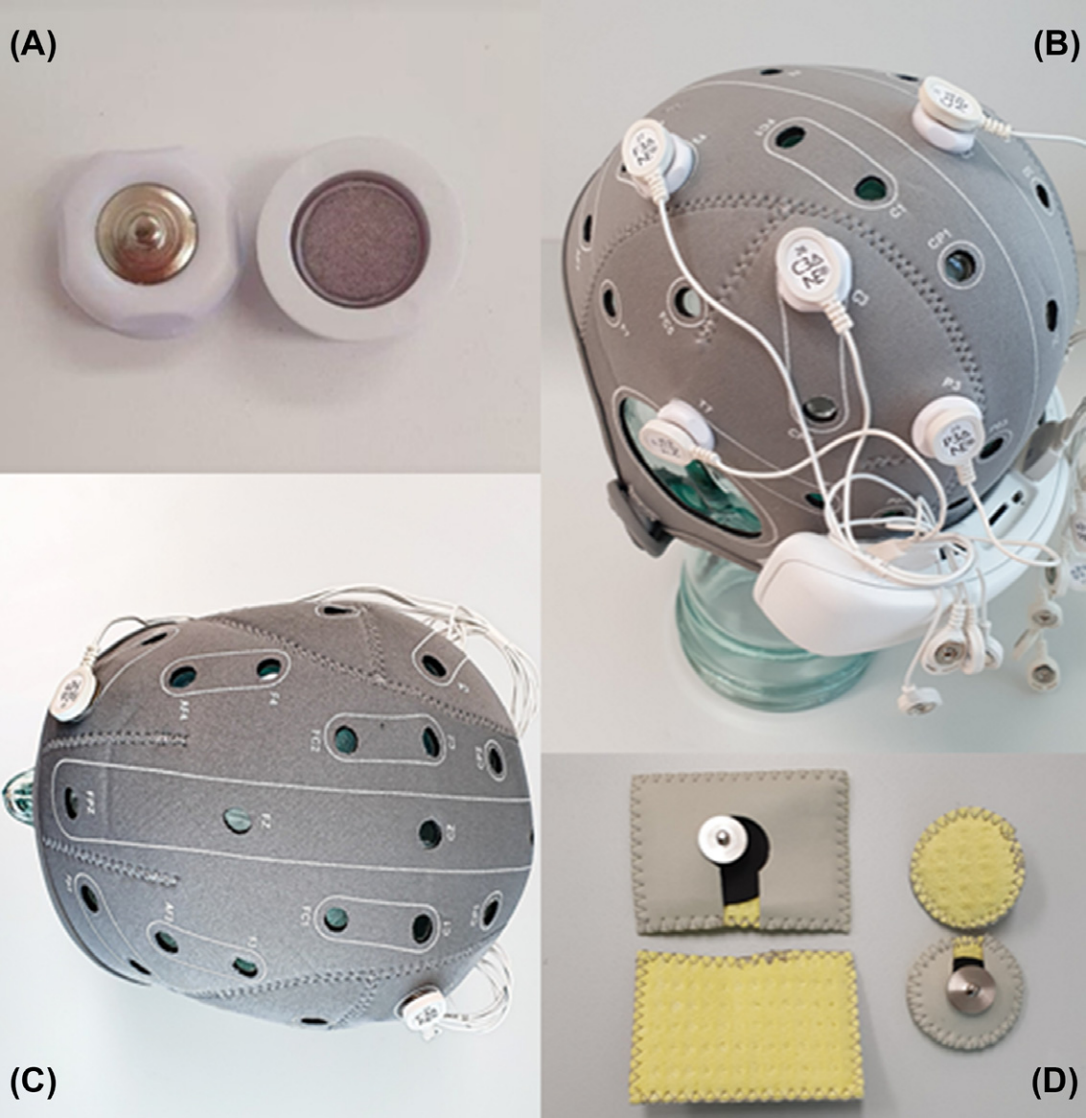
(A) High-definition electrode. (B) High-definition configuration, an active high-definition electrode is surrounded by four return HD electrodes. Stimulation could be anodal or cathodal depending on the direction of the applied current. (C) Conventional configuration, the stimulation is used with two active and return sponge electrodes (D). The stimulation is called anodal if the active electrode is above the targeted area and is called cathodal if the electrode placement is vice versa.

**Figure 2:**
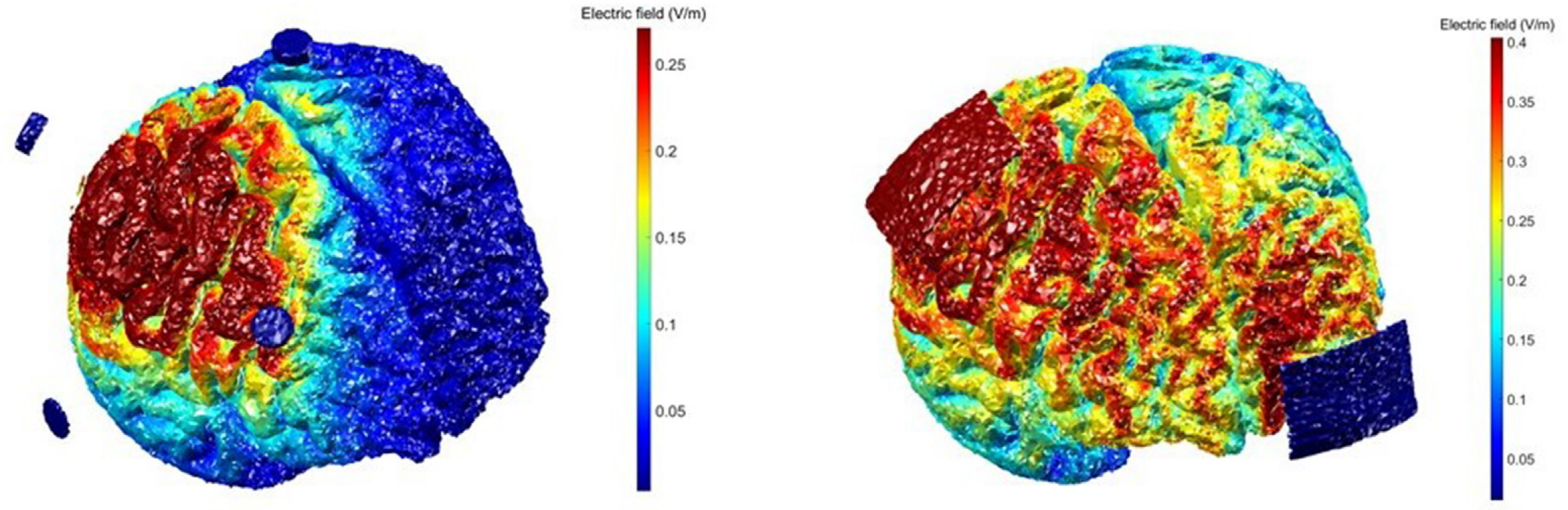
Simulation of the electric field generated by the stimulation of M1 with the application of a 2-mA direct current. As shown, a more focal electric field is produced with use of HD electrodes (left image) compared with the sponge electrodes (right image). The images were created by running simulations using the realistic volumetric approach to simulate transcranial electric stimulation (Huang *et al*., 2016; Huang *et al*., 2019).

The tDCS electrodes can be configured in two different methods: conventional and focal (high definition, HD) montages. A conventional montage involves two large sponge electrodes. In the focal montage, the electrode types and their arrangement vary, where the most common positioning is surrounding an active electrode by four current-return electrodes (Villamar *et al*., 2013), see Fig. 1. The electrode placement and arrangement must be optimized based on targeted activation cortices. Based on the type of electrodes, the stimulation can be widespread over the skull or relatively restricted to a particular location, see Fig. 2. Either way, the stimulation is site specific, not site limited, which means the target of stimulation matters but the modulation is not exclusive to that specific spot (Costa *et al*., 2015). The conventional montage influences a wide area of the cortex, which makes it difficult to associate stimulation results with the alteration of any specific part of the brain. With focalized stimulation, the small electrodes are used to stimulate specific regions of the cortex (DaSilva *et al*., 2011). Therefore, the HD montage potentially allows for targeting specific areas of the brain such as the sensory and motor cortex separately.

The effect of tDCS is not as large as TMS when it comes to the strength of facilitation (Brunoni *et al*., 2012); however, studies have shown that tDCS can be effective in alleviating pain (Brunoni *et al*., 2012; Costa *et al*., 2015; Lefaucheur, 2016) and also as a tool to investigate brain mechanisms (Bocci *et al*., 2015, 2018, 2019) (albeit, the functional mechanism of tDCS itself is not clearly understood). Based on current findings, there are two main hypotheses:

***Hypothesis 1: influencing the activation threshold of stimulated neurons***

**Figure 3:**
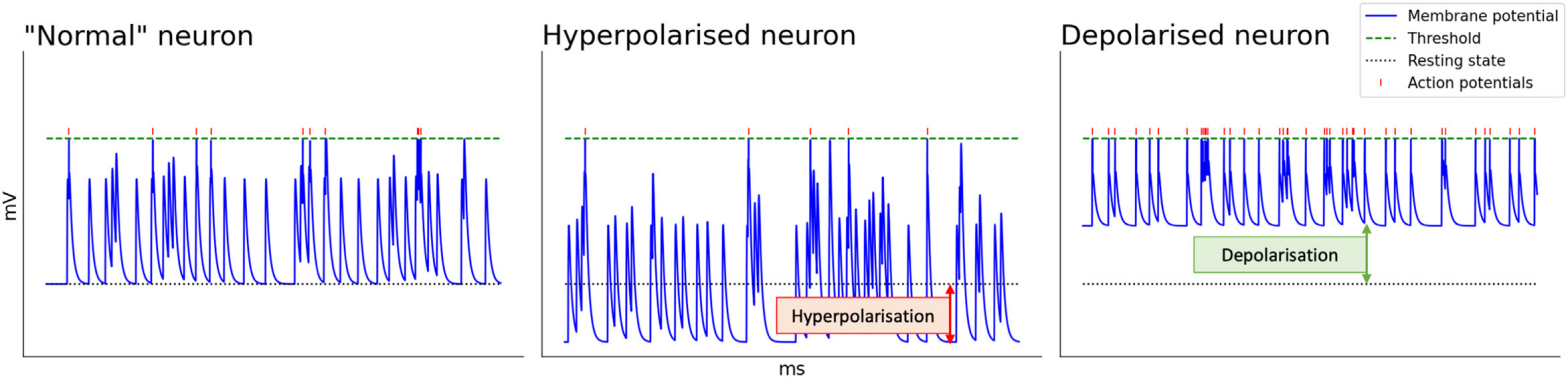
Images from left to right show the normal neuron activity, a hyperpolarized neuron as the result of cathodal tDCS, and a depolarized neuron as the result of anodal tDCS, respectively. The images were created with the Brian 2 simulator for spiking neural networks (Stimberg *et al*., 2019).

**Figure 4:**
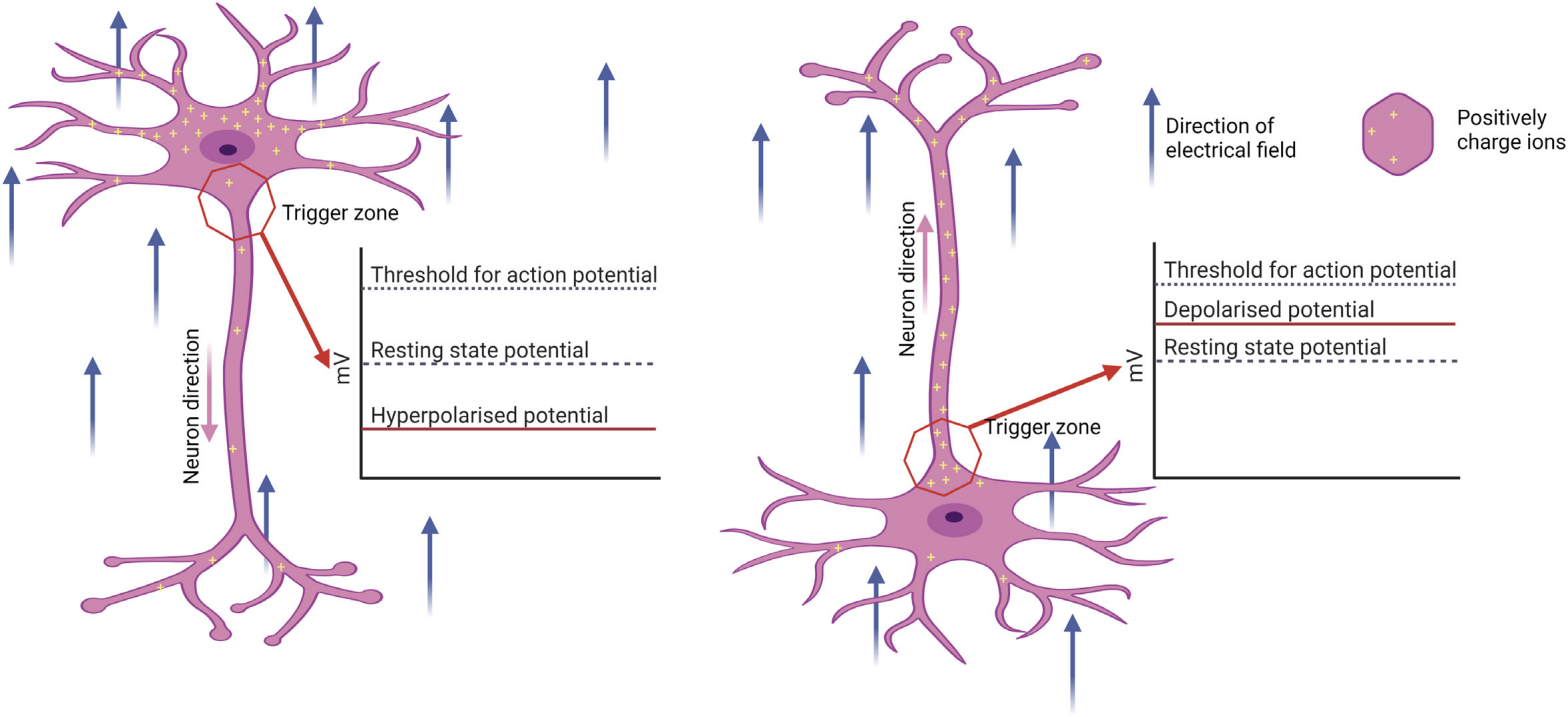
The illustration shows a single neuron under the influence of electric field generated by tDCS. Depending on the relative position of the axonal orientation and the direction of the electric field, the neuron hyperpolarizes (left picture) or depolarizes (right picture) (Kabakov *et al*., 2012; Kandel, 2021). The image was created with BioRender.com.

Cortical reorganization can be interpretated as two different phenomena: reorganization in cortical mapping (somatotopic organization), and changes in the somatosensory and motor cortices’ excitability (Knotkova and Cruciani, 2006). Studies have shown that brain modulation alters the latter (cortical excitability), which has arguably lead to pain reduction in patients with neuropathic pain (Inukai *et al*., 2016). Compared with other brain modulation techniques such as TMS, the field induced by tDCS is too weak to induce action potentials. With tDCS, the aim instead is to modulate the resting membrane potentials of neurons, and thereby also their excitability and spontaneous activity (Stagg *et al*., 2018). tDCS is polarity dependent, which means that once a cell is stimulated, the resting membrane potential either moves in the direction of depolarization, which means that less synaptic input is required for an action potential to be induced, or in the direction of hyperpolarization, in which the excitability is reduced, see Fig. 3. Furthermore, the efficacy and directionality of the stimulation is highly dependent on the orientation of the neurons relative to the electric field. The efficacy is greatest when the neuron axis aligns with the electrical field, and smallest when the two are perpendicular (Stagg *et al*., 2018). Neurons oriented at 0° and 180° to the applied electrical field will be depolarized (Fig. 4 right) and hyperpolarized (Fig. 4 left), respectively. These directional effects mean that the modulatory effect of tDCS will vary depending on the placement of the electrodes and the alignment of neurons in the targeted region (Stagg *et al*., 2018), see Fig. 4.

The effect may also vary depending on the stimulation intensity and duration. Batsikadze *et al*. compared anodal and cathodal tDCS on the primary motor cortex (M1) at currents of 1 and 2 mA (Batsikadze *et al*., 2013). Motor evoked potentials were measured to evaluate changes in motor corticospinal excitability. Their results revealed that both anodal and cathodal stimulation at 2 mA increased the corticospinal excitability, while cathodal stimulation at 1 mA decreased it (Batsikadze *et al*., 2013).

***Hypothesis 2: alterations in synaptic microenvironment potentially leading to longer lasting plastic changes***

The effects of tDCS occur during stimulation and generally do not carry over once stimulation is terminated when the stimulation sessions are few and short (e.g. a single session of few seconds). However, after longer-lasting stimulation periods (e.g. five sessions of 20 minutes each) other effects can be observed. In 2002, Liebetanz *et al*. studied tDCS alone and in combination with a Na^+^-channel blocker (carbamazepine) or a *N*-methyl-D-aspartate (NMDA) receptor antagonist (dextromethorphan) (Liebetanz *et al*., 2002). In the controlled drug-free condition, cortical excitability increased up to 40% with anodal stimulation. The Na^+^-channel blocker selectively eliminated the anodal (excitatory) effects, revealing that facilitatory aftereffects demand depolarization of the membrane potentials. On the other hand, with application of the NMDA-receptor antagonist, the aftereffect of stimulation was eliminated in both the case of anodal and cathodal stimulation. This result indicates that NMDA receptors are involved in tDCS-induced neuroplasticity (Liebetanz *et al*., 2002). With the activation of NMDA receptors, Ca^2+^ influx increases. High and low rates of Ca^2+^ influx lead to long-term potentiation (LTP) and long-term depression (LTD), respectively, and there is a transition zone where the influx of Ca^2+^ does not induce either LTP or LTD (Stagg *et al*., 2018).

Furthermore, magnetic resonance spectroscopy studies have showed altered glutamate and gamma-aminobutyric acid levels after tDCS (Stagg *et al*., 2009). Glutamate and gamma-aminobutyric acid are neurotransmitters involved in the synaptic plasticity, LTP, and LTD mechanisms.

## tDCS as a Treatment for PLP

Independent from physical therapy and psychological interventions, brain modulation by applying tDCS over different brain cortices has been used as a treatment to alleviate PLP (Bolognini *et al*., 2013; Bolognini *et al*., 2015). However, it is possible that integration of brain modulation and physical therapy could further reduce pain (Ortiz-Catalan, 2018) compared to applying each technique separately. Brain stimulation alone (Bolognini *et al*., 2015) and combined with physical therapy, including mirror therapy (Pinto *et al*., 2016; Boone and Frey, 2019; Ferreira *et al*., 2020; Gunduz *et al*., 2021), motor therapy (Kikkert *et al*., 2019), and motor imagery (Pacheco-Barrios *et al*., 2021), has been investigated in recent years. We summarize the outcomes of this research in Table 1.

**Table 1:** Investigational studies on PLP with the application of tDCS.

	Study design	Number of participants	Site of anodal electrode(s)	Site of cathodal electrode	Type of electrodes	Simulation intensity (mA): polarity	Number of sessions/duration of each session
(Bolognini *et al*., 2013)	RCT–tDCS vs sham	8	M1	Supraorbital area	35 cm^2^ sponge electrodes	2 anodal	2/15 mins
		7	PPC	Supraorbital area	35 cm^2^ sponge electrodes	2 anodal	2/15 mins
(Bolognini *et al*., 2013)	CO–double blinded	1	M1	Supraorbital area	35 cm^2^ sponge electrodes	2 anodal	5/15 mins
(Bolognini *et al*., 2015)	CO–double blinded	8	M1	Supraorbital area	35 cm^2^ sponge electrodes	1.5 anodal	10/15 mins
(Pinto *et al*., 2016; Gunduz *et al*., 2021)	RCT–tDCS and mirror therapy	112	M1	Supraorbital area	35 cm^2^ sponge electrodes	2 anodal	10/20 mins
(Kikkert *et al*., 2019)	tDCS–task concurrent, 4 experiments	17	S1/M1	Supraorbital area	35 cm^2^ sponge electrodes	1 anodal	1 per experiment/20 mins
(Bocci *et al*., 2018)	tDCS	8	Cerebellum	–	–	2 anodal	5/20 mins
(Bocci *et al*., 2019)	CO	14	2 cm below inion- midline	Right shoulder	35 cm^2^ sponge electrodes	2 anodal	5/20 mins
(Boone and Frey, 2019)	Case study– tDCS followed by mirror therapy	1	M1	Supraorbital area	35 cm^2^ sponge electrodes	1.5 anodal	10/20 mins
(Segal *et al*., 2020)	RCT–double blinded	30	M1	Forehead	35 cm^2^ sponge electrodes	1.5 anodal	10/22 mins
(Ferreira *et al*., 2020)	RCT–pilot study–tDCS and mirror therapy	16	M1	Supraorbital area	25 cm^2^ sponge electrodes	2 mA	12/30 mins
(Pacheco-Barrios *et al*., 2021)	Single arm protocol–tDCS and motor imagery	10	M1	Supraorbital area	35 cm^2^ sponge electrodes	2 mA	20/20 mins

RCT: randomized controlled trial, CO: crossover, S1/M1: sensorimotor cortex, hemisphere of anode (active electrode): contralateral to the side of amputation, hemisphere of cathode (return electrode): ipsilateral to the side of the amputation, Saline-soaked sponge electrodes.

Several parameters such as site of stimulation, current intensity, type of electrode, and polarity have varied in brain modulation studies with tDCS. Overall, these studies have shown that stimulation over M1, dorsolateral prefrontal cortex, posterior parietal cortex (PPC), and cerebellum can reduce pain (Lefaucheur *et al*., 2008; Lefaucheur, 2016).

Bolognini *et al*. conducted one of the first explorations on the management of PLP using tDCS (Bolognini *et al*., 2013). They tested the effect of a single session of anodal versus sham stimulation over M1 in a randomized crossover trial on eight participants (Bolognini *et al*., 2013). The study showed short-lasting analgesic effects of anodal tDCS on PLP for up to 90 minutes after stimulation. Following that, the same group evaluated the effect of five consecutive sessions of anodal tDCS over M1 as a single case study (Bolognini *et al*., 2013), and later in a larger group of eight participants (Bolognini *et al*., 2015). Their results were line with their earlier study and the followups in this second study revealed a five-times longer-lasting effect, arguably as a result of receiving a greater number of interventions.

The analgesic effect of a single session of tDCS over sensorimotor S1/M1 combined with phantom movement was examined and observed by Kikkert *et al*. (2019) on 17 individuals suffering from PLP in a randomized controlled double-blind study. Boone and Frey (2019) conducted a case study to evaluate the analgesic effect of applying 10 consecutive sessions of anodal tDCS over M1 followed by mirror therapy. At 1 week followup, pain reduction in average daily PLP was observed. Later, another randomized controlled double-blinded study by Segal *et al*. (2020) aimed to assess whether integration of mirror therapy with anodal tDCS over M1 increases the analgesic effect of mirror therapy in individuals with PLP. A higher pain reduction was observed in the group of participants who received both mirror therapy and active anodal tDCS, compared to the other two groups who received either mirror therapy alone or mirror therapy combined with sham tDCS. These findings were also supported by another pilot randomized controlled double-blinded study (Ferreira *et al*., 2020), in which it was observed that the combination of mirror therapy with anodal tDCS over M1 had a stronger analgesic effect than mirror therapy combined with sham tDCS, albeit in the treatment of neuropathic pain due to brachial plexus avulsion. However, the results of a larger randomized controlled clinical trial (Pinto *et al*., 2016; Gunduz *et al*., 2021), with the aim of comparing the effects of four possible combinations of active/covered mirror therapy and active/sham anodal tDCS over M1, showed that the effects of anodal tDCS over M1 and mirror therapy on PLP are independent and they found that only active anodal tDCS has a statistically significant effect on PLP alleviation. Last, an ongoing single arm study, by Pacheco-Barrios *et al*., is investigating the feasibility of a home-based combined treatment, constituting tDCS and motor imagery, for a larger remote trial (Pacheco-Barrios *et al*., 2021).

Regarding other brain areas, the influence of a single session of anodal and cathodal stimulation over PPC in seven participants with limb amputation was studied by Bolognini *et al*. (2013). They found that the hyperpolarization of PPC concluded in reduction of nonpainful phantom sensation, and neither excitation nor inhibition of PPC affected PLP or stump pain. Therefore, no correlation between the activation of PPC and reduction of PLP was observed.

The potential role of cerebellar tDCS in pain perception has been proposed by Bocci *et al*. (2015). Bocci *et al*. investigated the modulatory effect on PLP of cerebellar tDCS and concluded that the anodal tDCS, compared with sham tDCS, improved the paroxysmal pain (episodes of increased PLP) and nonpainful phantom sensation, but not the constant PLP (Bocci *et al*., 2018). One year later, Bocci *et al*. conducted a crossover, double-blind, sham-controlled clinical trial, with a similar protocol to the previous study, to compare the impact of the anodal, cathodal, and sham cerebellar tDCS on PLP. The results supported the earlier study and, furthermore, reduction in the phantom movement from stimulation of anodal polarity was observed (Bocci *et al*., 2019).

Limited studies have examined the effect of focalized stimulation with high-density tDCS (HD-tDCS) on pain. A study by Borckardt *et al*. found a reduction in cold and heat sensory thresholds, reduction in thermal windup pain, and mild changes to cold pain thresholds in 24 healthy participants (Borckardt *et al*., 2012). Furthermore, in another study by Villamar *et al*., reduction in overall pain perception was observed in individuals with fibromyalgia (Villamar *et al*., 2013). Although we found no studies on HD-tDCS used to treat PLP in the literature, the early studies mentioned previously indicate that this might be an alternative modality worthy of investigation.

## tDCS as a Neuroscientific Tool for Investigating PLP

NIBS techniques have been used as investigational tools to explore the role of the different cortices of the brain. In particular, tDCS has been applied in many studies to investigate correlations between changes in brain activity and various brain conditions including neurological disease, mental illnesses, and brain disorders (Lippold and Redfearn, 1964; Hummel *et al*., 2005; Lefaucheur, 2016).

Regarding PLP, the exact mechanism underlying the condition is yet unknown (Di Pino *et al*., 2021). Many studies have shown correlations between PLP and reorganization of sensory and motor cortices, however, these results are not conclusive (Andoh *et al*., 2020). Flor *et al*. showed that displacement of adjacent regions into the region of the amputated limb in primary sensory cortex (S1) was positively correlated with the intensity of PLP (Flor *et al*., 1995). The same group later demonstrated that less intense PLP was correlated with more activity in the sensorimotor cortex during phantom motor imagery together with mirrored movement of the contralateral, intact limb (Diers *et al*., 2010). On the other hand, Kikkert *et al*. found a positive correlation between PLP and activity in the affected sensorimotor cortex (Kikkert *et al*., 2019). In a recent study, Andoh *et al*. found that these seemingly contradictory findings, at least in part, could be explained by differences in defining and analyzing regions of interest in functional magnetic resonance imaging (fMRI) data (Andoh *et al*., 2020). The results from this study also suggest that sensory and motor maps differentially relate to PLP. It must also be taken into consideration that the studies mentioned have only been able to show correlation, not causation. Thus, the cortical reorganization could in fact be a result of other processes that are the actual drivers of PLP (Ortiz-Catalan, 2018). More importantly, studies on the sensorimotor cortex have provided no direct relation to pain processing and how changes in this part of the brain could maintain or initiate PLP (Ortiz-Catalan, 2018).

Although tDCS is limited to neurons in the most superficial regions of the brain, the method by itself, or integrated with behavioral tasks, could still serve as a useful tool in investigating possible deeper cortical and subcortical mechanisms related to PLP, potentially when used in combination with brain imagining [electroencephalography (EEG) or fMRI]. Ultimately, tDCS can enable the possibility of conducting double-blinded studies, as it supports sham conditions. An example of such a study was performed by Kikkert *et al*. and consisted of a double-blinded, sham-controlled trial stimulating M1 during phantom movements while simultaneously recording brain activity with fMRI (Kikkert *et al*., 2019). They found that reduced activity in sensorimotor cortex after stimulation was associated with pain reduction. This study also showed that the reduction in cortical activity was preceded by altered activity in the mid- and posterior insula and in the secondary somatosensory cortex. Phantom motor execution was unverified, and thus implementing the decoding of myoelectric signals could be a further improvement on this approach (Ortiz-Catalan *et al*., 2016; Ortiz-Catalan, 2018). More studies along these lines, with tDCS applied to different cortical regions in combination with brain imaging, could help elucidate the possible mechanism behind PLP. Furthermore, monitoring the more peripheral parts of the nervous system during and after stimulation could also shed light on the involvement of descending pain modulation.

## Conclusions

PLP is a complex medical condition that can be highly detrimental to the patients’ quality of life. PLP can be caused by injury at any level of the extremities and finding its underlying mechanisms is crucial for selecting optimal clinical treatments. The stochastic entanglement of the sensorimotor and pain processing networks has been hypothesized as the cause of PLP (Ortiz-Catalan, 2018), and treatments aiming to undo such entanglement by purposely activating sensorimotor networks have shown promising results (Ortiz-Catalan *et al*., 2016). Outcomes from such therapies could be further improved using brain modulation, for instance by facilitating motor learning in case of PLP by anodal tDCS (Pan *et al*., 2015). In particular, a mostly conventional montage of tDCS has been used as an investigational tool for understanding brain mechanisms and as a method of treatment of various brain conditions and neurological disorders. However, there are limited studies on its application on PLP and therefore its working mechanism as a treatment. So far, it has been hypothesized that depolarization of the affected sensorimotor cortex by anodal tDCS reduces PLP, however, how depolarization causes PLP reduction has not yet been understood.

Studies have nevertheless shown that conventional anodal tDCS over the affected M1 alone or integrated with other therapies has an analgesic effect on PLP, but more effectively designed randomized controlled clinical trials with sufficiently large numbers of participants are lacking. Therefore, larger and more rigorous studies, potentially using HD-tDCS and brain imaging techniques such as EEG or fMRI, could be highly beneficial in determining the efficacy of such approaches, and could possibly contribute to a better understanding of the pain mechanisms.
